# Minimally invasive stabilization using screws and cement for acetabular metastatic tumor: a case report

**DOI:** 10.1186/s13256-024-04604-1

**Published:** 2024-06-18

**Authors:** Yang Chen, Yunus Akbar, Haibin Xiang, Yashengjiang Yadikan, Guoqing Cao, Xiaowei Ju, Xiaoguang Wu, Shiwen Wang

**Affiliations:** 1 Department of Arthrology, 989th Hospital of PLA, Luoyang, 471000 Henan China; 2 Department of Bone Cancer, The First Affiliated Hospital of Xinjiang Medical University, Urumqi, 830000 Xinjiang China

**Keywords:** Acetabulum, Neoplasm metastasis, Fracture fixation, Internal, Orthopedic procedures

## Abstract

**Background:**

The aim of this case report is to evaluate minimally invasive stabilization using screws and cement for acetabular metastatic tumor and summarize the indications and contraindications for minimally invasive stabilization of acetabular metastatic tumors with screw and cement techniques.

**Case presentation:**

Under imaging guidance, a patient with acetabular metastatic tumor was treated with hollow screw combined with bone cement fixation. Ischial screw, ascending branch screw, and anterior and posterior screws were inserted to firmly fix the anterior and posterior column of the acetabulum. At the same time, the third screw connected the anterior and posterior columns together, combined with bone cement into the fracture site to further increase local stability and resist bone defects caused by local tumor osteolysis. The patient was a 52-year-old Uygur male. Herein, we summarize his clinical symptoms and operation. Differences in visual analog scale and walking function (Musculoskeletal Tumor Society) before operation and at 2 months, 6 months, and 12 months after operation were compared.

**Results:**

Postoperative complications and tumor progression were recorded. The patient was followed up for 16 months, and the operative time was 60 minutes. In total, 20 ml of bone cement was injected into the acetabular posterior column and the top of the acetabulum. VIsual analog scale score was 8 before operation, 3 at 2 months, 3 at 6 months, and 2 at 12 months after operation. Musculoskeletal Tumor Society function was 13 before operation, 23 at 2 months, 25 at 6 months, and 26 at 12 months after operation. During follow-up, no cement leakage, fever, hip nerve injury, pulmonary embolism, or imaging findings of further destruction of the acetabulum and surrounding bone were noted.

**Conclusion:**

This case report shows that the treatment of acetabular metastatic cancer with minimally invasive stabilization using screws and cement under the C arm can effectively relieve pain and enhance the strength of the pelvis, and is innovative and feasible.

## Introduction

For bone metastatic tumors, it is estimated that there are as many as 100,000 patients every year, among which the bone metastasis rate of breast and prostate cancer is the most significant, accounting for 75% of the total number of bone metastatic tumors, and 40% of patients with bladder and lung cancer [1]. In bone metastatic cancer, because of the great pressure in the process of weight-bearing in the pelvic region, the problem of pelvic metastasis is particularly serious [2], especially in the parts with complex anatomical structure, such as the acetabulum, which is very difficult to remove and reconstruct. At present, there is no best treatment [3]. For metastatic tumors in the pelvis, a wide range of Harrington reconstruction methods are traditionally needed to reconstruct the acetabulum, including tumor resection, prosthesis reconstruction at pelvic defects, etc. [4]. However, this kind of major operation has a high incidence of postoperative complications, and it takes a long time for postoperative functional recovery; at the same time, the recovery time of incision in the operative area is longer, delayed or interrupted by the radiotherapy and chemotherapy of metastatic tumors, and further delays the systematic treatment of primary tumors [5, 6]. Therefore, for patients with acetabular metastatic tumors, especially those with severe pain and pelvic stability, minimally invasive stabilization using screws and cement can not only obtain good local stability and relieve pain symptoms but also enable patients to receive systematic treatment earlier and prevent tumor progression to a certain extent.

## Clinical data

The patient, a 52-year-old Uygur male, was admitted to hospital because of “pain in the proximal left thigh for more than 3 months, aggravated with dyskinesia for 4 days.” At 3 months ago, the patient reported that he had pain in the proximal left thigh after excessive activity, which was aggravated during exercise and relieved during rest. During that time, he received traditional Chinese medicine treatment in the local hospital. Then, 4 days ago, the pain in the left hip was aggravated owing to a fall, and the activity of the left lower limb was limited owing to pain. He was immediately referred to our hospital by the local hospital. Physical examination showed that the skin of the left acetabulum was blue, the tenderness was obvious, and the activity of the left hip joint was obviously limited because of the pain.

### Preoperative examination

**Fig. 1:**
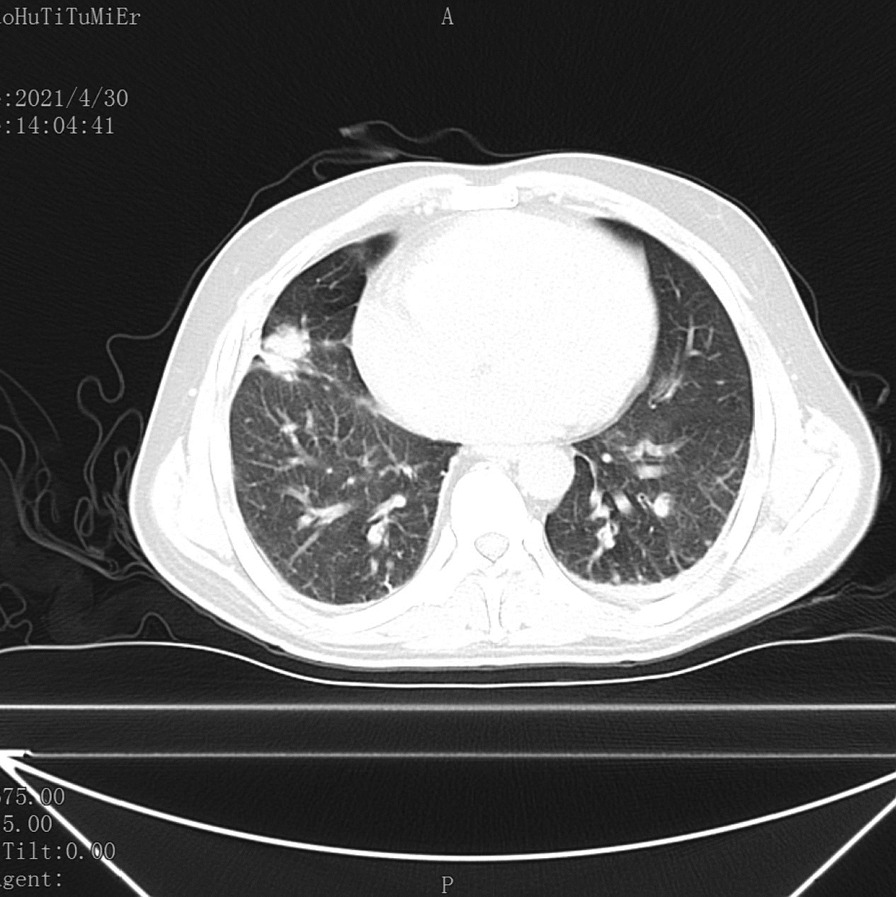
Preoperative lung Computed tomography (CT) showed a round mass in the middle lobe of the right lung, and the primary lesion was considered to be lung cancer

X-ray examination showed that the bone density of the left ischial bone and acetabulum decreased inhomogeneously, slightly expanded locally, and the ischial, pubic, and acetabular bones were discontinuous. The left acetabular tumor was considered as venereal disease with pathological fracture. Computed tomography (CT) suggested that the alignment of the left hip joint was poor, the left ischial, pubic, and acetabular cortex was not continuous, and the soft tissue density could be seen; the lung CT suggested that a kind of round soft tissue density mass could be seen in the middle lobe of the right lung, the margin was lobulated, fibrous cord could be seen around it, and pleural tension between the adjacent lobes was about 2.17 cm × 1.93 cm, which was considered as a possible peripheral lung cancer (Fig. 1). Magnetic resonance imaging (MRI) suggested that the signal intensity of left ischial, pubic, and acetabular bone was discontinuous and multiple bone fragments could be seen. Patchy T1 and slightly longer T2 signal could be seen in the broken bone of the fracture, hyperintensity was seen in fat compression sequence, irregular mass in soft tissue was seen in T1 mixed with long T2 signal, fat compression sequence showed mixed and slightly high signal intensity, and uneven enhancement was observed after enhanced scan. The results of preoperative electrocardiogram (ECG) showed that there were multiple active foci of bone metabolism, which was considered as malignant metastasis.

### Preoperative design

**Fig. 2:**
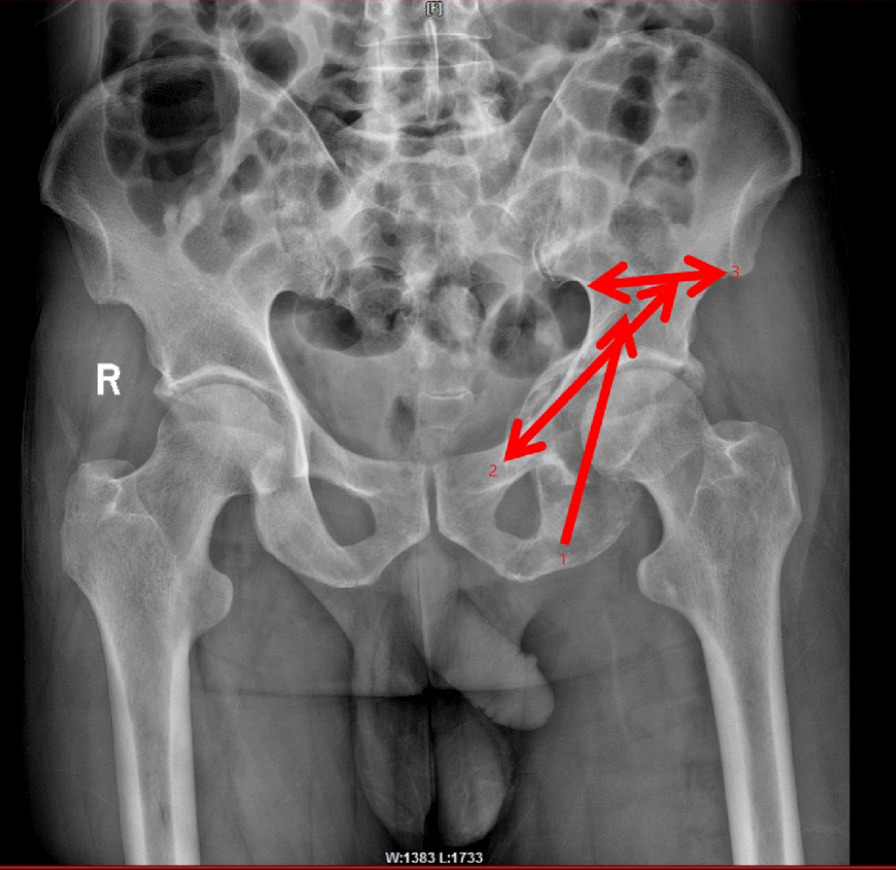
Preoperative design (arrow 1 represents the ischial nail to the iliac spine through the ischial tubercle; arrow 2 represents the ascending branch screw from the anterior inferior iliac spine to the superior pubic branch; arrow 3 represents the anterior and posterior screws from the anterior inferior spine to the posterior inferior spine)

The patient is a middle-aged male; combined with the results of ECG and pulmonary CT examination, the preliminary diagnosis of lung cancer bone metastasis with pathological fracture was made. Considering that the patient has multiple bone lesions, not a single metastasis, and the main clinical symptoms are hip pain and limited movement, conservative treatment would be ineffective; if the expected survival time is not less than 3 months, it is necessary to receive systematic treatment for the primary focus as soon as possible. Preoperatively, the relevant examination was improved to exclude contraindications to general anesthesia and rule out a history of bone cement allergy in accordance with the surgical indications of our department. Additionally, the ischial screw, ascending branch screw, and anterior and posterior screws were designed before the operation (Fig. 2). The direction of each screw was defined under C-arm, and the puncture needle was inserted through the screw direction to establish a working channel casing and inject bone cement into the fracture site.

### Surgical procedure

After the anesthesia was effective, the patient was placed in the right recumbent position and the ischial tubercle and anterior inferior iliac spine were marked under the C-arm. A 0.5-cm longitudinal incision was made at the location of the ischial tubercle. Under the guidance of the C-arm, the needle was inserted at the lateral side of the ischial tubercle toward the anterior 1/3 marked point of the iliac crest and passed through the posterior column of the acetabulum and the fracture line of the ischium. The Kirschner needle passed through the posterior column of the acetabulum and did not pass through the hip joint in the oblique position of the ilium and the frog position of the acetabulum. The longitudinal incision at the location of the anterior inferior iliac spine on the affected side was about 0.5 cm. Under the guidance of the C-arm, the needle was inserted in the direction of the anterior inferior iliac spine toward the posterior inferior iliac spine, and assessed to ensure that the Kirschner needle was located above the acetabulum. Under the guidance of the C-arm, the needle was inserted toward the superior branch of the pubis, 2 cm from the anterior inferior iliac spine of the affected side. The oblique obturator and frog position of the acetabulum showed that the Kirschner needle passed through the anterior column of the acetabulum and did not pass through the hip joint. After ensuring that the hip joint of the affected side did not touch the obvious clamping, proceed along the puncture needle. A hollow drill was used, the hollow screw was then inserted in the direction of the puncture needle, and after screw fixation, the C-arm was used again to ensure that the screw did not pass through the hip joint and through the fracture line. The puncture needle was inserted along the direction of the screw at the ischial tubercle and the anterior inferior iliac spine, the working channel was established, the ring drill was inserted, and bone tissue was taken for pathological examination. Under continuous fluoroscopy, bone cement was slowly injected into the acetabular posterior column and the top of the acetabulum through the upper and lower working channels, and 20 ml of bone cement was injected into the upper and lower sides. Under C-arm fluoroscopy, it was seen that the bone cement was filled and there was no bone cement overflow at the lesion site of the acetabular posterior column. After the bone cement solidified, the working casing was removed. Suture and bandaging were performed after washing.

### Postoperative follow-up

**Fig. 3:**
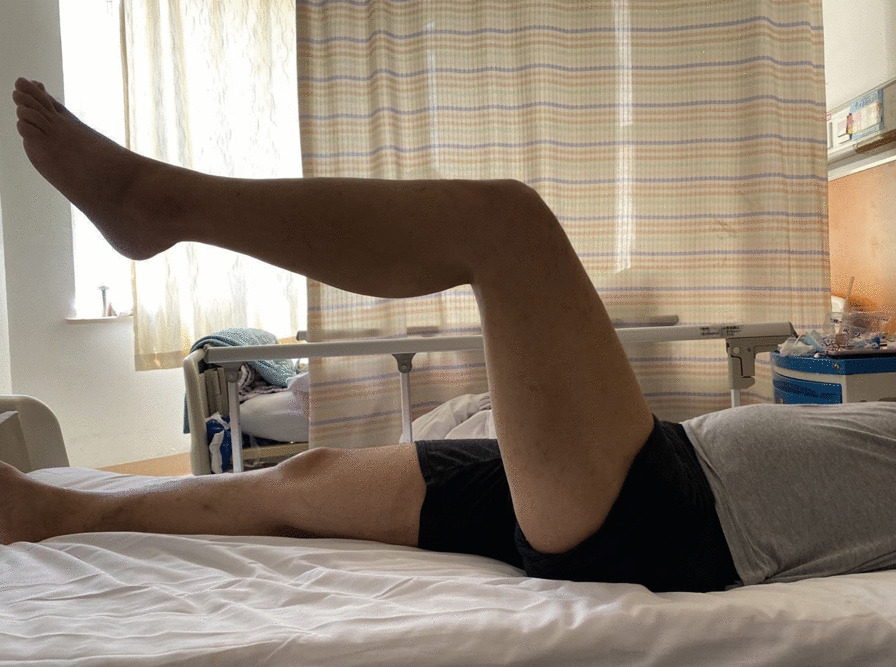
At 2 months after operation, the limb activity of the patient show that the function of the affected limb is basically normal

**Fig. 4:**
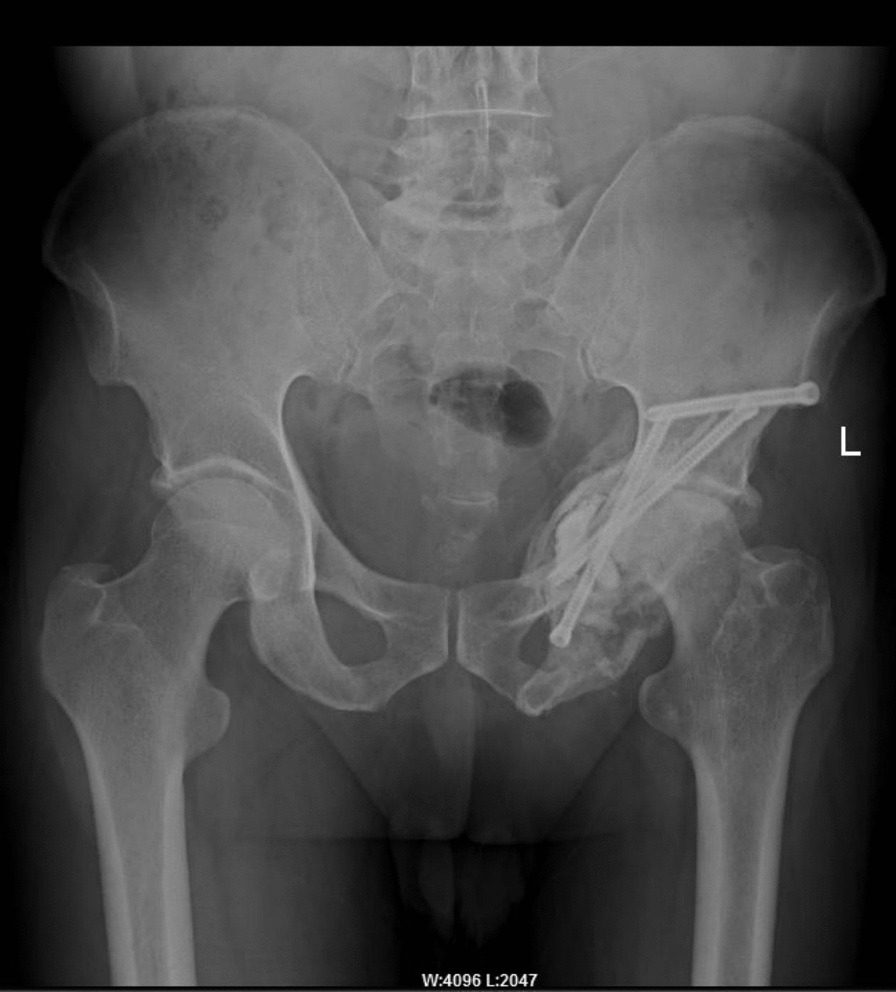
At 13 months after operation, the positive position of the pelvis show that the screw cement is in good position, and no further destruction of acetabulum and surrounding bone can be found

At 12 hours after the operation, the patient reported that the hip pain was improved, and joint movement could be performed on the bed. On the second day, the patient could move on the ground with the assistance of a brace. At 1 week after the operation, the oncology department confirmed lung adenocarcinoma by percutaneous lung biopsy, and mutation of L858R gene was detected by next generation sequencing (NGS). Oxitinib was given as a targeted treatment. After excluding the contraindications of radiotherapy, the radiotherapy plan was made: clinical target volume (CTV): left ischia, pubis, acetabulum, tumor absorbed dose (DT): 45 Gy/2.5 Gy/18F, planning target volume (PTV): CTV + 0.7 CM, DT: 45 Gy/2.5 Gy/18F. At 2 months after the operation, the hip pain was significantly improved an the follow-up, hip flexion was 0–90°, external rotation was 0–40°, abduction was 0–45°, the patient was able to move using crutches, and weight-bearing of the affected limb gradually increased until finally it was gradually removed from the brace (Fig. 3). At 13 months after operation, the results of positron emission tomography-CT (PET-CT) showed that there was no further progress in the local and pulmonary hypermetabolic lesions in the operative area (Fig. 4), and the metabolism of the left frontal lobe was abnormal, which was considered as metastatic focus. The combination of chemotherapy and targeted therapy was performed: pemetrexed + nedaplatin + bevacizumab intravenous, oral oxetinib targeted therapy, and radiotherapy regimen at 54 Gy/3 Gy/18F for brain lesions. Using the pain visual analog scale (VAS), a score of 0 represents no pain, and the 10 indicates the most severe pain. In the functional follow-up, the lower limb function was evaluated according to the functional reconstruction evaluation standard of bone and soft tissue tumor established by the International Association of Bone Oncology [7] (Musculoskeletal Tumor Society, MSTS). The highest score was 30, with a score of 5 points in each aspect from six aspects: pain, function, psychological tolerance, support, walking function, and gait. Follow-up results were as follows: VAS score was 8 before operation, 3 at 2 months, 3 at 6 months, and 2 at 12 months after operation; MSTS function score was 13 before operation, 23 at 2 months, 25 at 6 months, and 26 at 12 months after operation. Pain relief and functional recovery were satisfactory. As of the last follow-up, there was no imaging manifestation of further destruction of acetabulum and surrounding bone. This case report shows that the treatment of acetabular metastatic cancer with minimally invasive stabilization using screws and cement under the C arm can effectively relieve pain and enhance the strength of the pelvis, which is innovative and feasible.

In summary, this case report shows that the treatment of acetabular metastatic cancer with minimally invasive stabilization using screws and cement under the C arm can effectively relieve pain and enhance the strength of the pelvis, which is innovative and feasible.

## Discussion and conclusion

### Surgical treatment of acetabular metastatic tumors

According to the Enneking pelvic tumor zoning standard [8], periacetabular tumors belong to zone II tumors, which usually undergo type II pelvic resection, which is of great significance for the treatment of primary acetabular malignant tumors. However, for metastatic tumors, there is no direct relationship between acetabular resection and survival rate [9]. According to the Harrington [10] grading standard of metastatic bone destruction around the acetabulum, grade I is periacetabular bone destruction, but the acetabular wall bone cortex is intact, grade II is acetabular bottom bone destruction and defect, and grade III is acetabular bone defect. Harrington [10] and Stark *et al*. [11] performed total hip arthroplasty with or without acetabular support ring to reconstruct function for grade I and II metastatic bone defects around the acetabulum. Guo *et al*. [9] assessed grade I bone destruction in area I and II, and found that the pain was obviously relieved, the pelvis was stable and the function of hip joint was well preserved by selecting nail-rod system or nail-rod system combined with bone cement fixation and reconstruction. For grade II acetabular defect, focus curettage, bone cement filling, and acetabular metal reinforced mesh cup combined with artificial joint replacement were used to obtain satisfactory hip joint stability and good functional recovery. The patient in this study belonged to grade II bone destruction in area II and area III, but the patient did not undergo reconstruction by artificial joint replacement because of the following reasons: comprehensive evaluation of the patient’s condition, the preliminary diagnosis was bone metastasis of lung cancer with multiple bone lesions in the whole body. In addition to the short-term treatment goal of relieving clinical symptoms, patients also need to start systemic treatment as soon as possible to delay the progression of the disease. If artificial joint replacement is selected, the surgical trauma is greater, the risk of infection after intraoperative implantation of prosthesis is increased, and incision healing and postoperative rehabilitation exercise are delayed for a large amount of time, which will inevitably delay systemic treatment. Therefore, we believe that the choice of treatment should not be limited to the severity of pain, limited activity, quality of life, and so on, but also needs to integrate the life expectancy of patients, whether it is metastatic disease and other site metastasis.

### Advantages of minimally invasive stabilization using screws and cement technology

The technique of minimally invasive stabilization using screws and cement can be used as an optimal operation for patients who are not suitable for open surgery, are worried about complications, such as poor wound healing, need continuous systemic treatment, or have rapidly progressive diseases. This operation has the following advantages: (1) the combined application of hollow screw and bone cement; because the acetabulum is the key part of human body weight-bearing, the bone destruction caused by metastatic tumor will lead to the decrease of the mechanical properties of acetabular bone, resulting in pathological fracture and affecting the function of hip joint. Thus, in treatment, the application of bone cement can resist the pressure of large osteolytic defects, especially in the top area of acetabulum, because it exerts the most significant force in normal activities. Secondly, bone cement helps to further stabilize the screws in the area of bone destruction, and the deposition of bone cement helps to prevent the progression of the disease to a certain extent, but there are some limitations in the treatment of grade II acetabular bone destruction only with bone cement. The bone cement will cause insufficient stability during joint rotation, bending, and movement, therefore increasing the screw can provide a more stable structure. In the selection of screws, we recommend to choose full-threaded hollow screws, because some of the screws will dynamically compress the fractured site and can be associated with vascular or nerve injury. At the same time, hollow screws can establish a working channel for biopsy and bone cement, which is more convenient for bone cement injection. (2) Through the establishment of working channel, the length of surgical incision can be reduced, the bleeding during operation is less, and the recovery after operation is quick. (3) This technical operation was completed under the guidance of C-arm. The position of C-arm was adjusted during the operation, and screws and bone cement were accurately placed under continuous fluoroscopy to ensure the success of the operation. (4) Postoperative local stability can be significantly increased, postoperative incision healing time can be reduced, and systemic treatment of primary tumor can be accepted earlier.

### Indications and contraindications for minimally invasive stabilization using screws and cement technology

Although the technique of minimally invasive stabilization using screws and cement can significantly enhance local stability, has a small incision, less bleeding during operation, and rapid recovery after operation, there are some shortcomings in the treatment of acetabular metastatic cancer, such as incomplete debridement and bone cement leakage, so the indications and contraindications should be strictly grasped. The indications for this treatment are: (1) patients with hip pain, limited movement of hip joint, unstable pelvis and poor conservative treatment; (2) patients who need systematic treatment as early as possible, in addition to acetabular metastatic lesions, accompanied by multiple lesions throughout the body; (3) patients whose expected survival time is not less than 3 months have certain requirements for quality of life; (4) for tumors that respond well to systemic and radiotherapy, such as breast cancer, prostate cancer, lung cancer, myeloma, and lymphoma, when acetabular metastasis occurs, unless accompanied by grade III bone destruction, this technique can be used to obtain satisfactory local stability, laying the groundwork for postoperative overall treatment. The contraindications are: (1) primary acetabular tumor or single acetabular metastasis; (2) patients with poor general condition and unable to tolerate general anesthesia; (3) a clear history of bone cement allergy; (4) expected survival time of no more than 3 months; (5) in patients with severe comminuted fracture or fracture with obvious displacement and acetabular edge fracture, screw placement is difficult and bone cement overflow is easy to occur during operation; (6) for tumors that are resistant to systemic and radiotherapy reactions, such as renal cell carcinoma and bladder cancer, this procedure can only achieve early stability and prevent bone-related events, but for holistic treatment, it is necessary for clinicians to formulate a satisfactory treatment plan according to the needs of patients.

At present, there are still many problems in the surgical treatment of pelvic metastases [8]. The focus is how to view the value of local surgery for the overall treatment and outcome of patients. The author believes that, for patients with pelvic metastases, if the minimally invasive stabilization using screws and cement technique is used for surgical treatment, it is necessary to strictly control the surgical indications and contraindications, and take local treatment as a part of the overall treatment. fully consider the life expectations of patients, and formulate a reasonable overall treatment plan.

In summary, this case report shows that the treatment of acetabular metastatic cancer with minimally invasive stabilization using screws and cement under the C arm can effectively relieve pain and enhance the strength of the pelvis, which is innovative and feasible.

## Data Availability

Not applicable.

## Abbreviations

VAS: Visual analog scale
MSTS: Musculoskeletal Tumor Society
CTV: Clinical target volume
PTV: Planning target volume
Gy: Gray
DT: Tumor absorbed dose
